# Genomes of the willow-galling sawflies *Euura lappo* and *Eupontania aestiva* (Hymenoptera: Tenthredinidae): a resource for research on ecological speciation, adaptation, and gall induction

**DOI:** 10.1093/g3journal/jkab094

**Published:** 2021-03-31

**Authors:** Craig Michell, Saskia Wutke, Manuel Aranda, Tommi Nyman

**Affiliations:** 1 Department of Environmental and Biological Sciences, University of Eastern Finland, Joensuu, 80100, Finland; 2 Biological and Environmental Sciences & Engineering Division, Red Sea Research Center, King Abdullah University of Science and Technology, Thuwal, 23955-6900, Saudi Arabia; 3 Department of Ecosystems in the Barents Region, Norwegian Institute of Bioeconomy Research, Svanvik, 9925, Norway

**Keywords:** genome, gall-inducing insects, sawfly, hybrid assembly

## Abstract

Hymenoptera is a hyperdiverse insect order represented by over 153,000 different species. As many hymenopteran species perform various crucial roles for our environments, such as pollination, herbivory, and parasitism, they are of high economic and ecological importance. There are 99 hymenopteran genomes in the NCBI database, yet only five are representative of the paraphyletic suborder Symphyta (sawflies, woodwasps, and horntails), while the rest represent the suborder Apocrita (bees, wasps, and ants). Here, using a combination of 10X Genomics linked-read sequencing, Oxford Nanopore long-read technology, and Illumina short-read data, we assembled the genomes of two willow-galling sawflies (Hymenoptera: Tenthredinidae: Nematinae: Euurina): the bud-galling species *Euura lappo* and the leaf-galling species *Eupontania aestiva*. The final assembly for *E. lappo* is 259.85 Mbp in size, with a contig N50 of 209.0 kbp and a BUSCO score of 93.5%. The *E. aestiva* genome is 222.23 Mbp in size, with a contig N50 of 49.7 kbp and a 90.2% complete BUSCO score. *De novo* annotation of repetitive elements showed that 27.45% of the genome was composed of repetitive elements in *E. lappo* and 16.89% in *E. aestiva*, which is a marked increase compared to previously published hymenopteran genomes. The genomes presented here provide a resource for inferring phylogenetic relationships among basal hymenopterans, comparative studies on host-related genomic adaptation in plant-feeding insects, and research on the mechanisms of plant manipulation by gall-inducing insects.

## Introduction

The hyperdiverse insect order Hymenoptera includes over 153,000 described species (Aguiar *et al.* 2013), but the true number may be 10 times higher (Forbes *et al.* 2018). Hymenopteran species have a multitude of important roles in our environment, including pollination, herbivory, and population control of other insects (Noriega *et al.* 2018). The high ecological and economic importance of hymenopterans has made many species and groups important model systems in theoretical and applied research.

The order Hymenoptera is divided into the ancestrally herbivorous, paraphyletic suborder “Symphyta” (sawflies, woodwasps, and horntails) and the ancestrally parasitic, monophyletic Apocrita (bees, ants, and wasps). Currently, there are 99 hymenopteran whole-genome assemblies present in the NCBI database (accessed February 2020). Only five of the available genomes represent hymenopteran lineages from the suborder Symphyta, while the remaining 94 belong to Apocrita. Although these numbers roughly correspond to the relative proportions of species in the two suborders, the uneven representation of genome-enabled hymenopteran taxa limits our possibilities for inferring phylogenetic relationships within the order (Branstetter *et al.* 2018) as well as genomic traits underlying shifts in niche use and rates of diversification (Oeyen *et al.* 2020). Fortunately, correcting the current bias should be relatively straightforward because hymenopterans are unusually accessible for whole-genome sequencing: hymenopteran genomes are generally small (the majority are between 180 and 340 Mbp) (Branstetter *et al.* 2018) and contain comparatively low rates of repetitive and transposable elements (Petersen *et al.* 2019). A further methodological benefit follows from their haplodiploid sex-determination system, which leads to the presence of haploid males, for which genome assembly is technically easier than for diploid individuals with intra-genomic sequence variation. Coupling these favorable genomic features with new sequencing technologies such as 10X Genomics linked-read sequencing and MinION ONT long-read sequencing, it is becoming easier to sequence and assemble high-quality genomes of these important insects.

The symphytan lineages of the Hymenoptera comprise 14 ancestrally herbivorous families and the parasitic sawfly family Orussidae (Nyman *et al.* 2019). In genome databases, symphytans are currently represented by the tenthredinid *Athalia rosae* (Oeyen *et al.* 2020), the diprionids *Neodiprion lecontei* (GenBank accession: GCA_001263575) and *Neodiprion pinetum* (GenBank accession: GCA_004916985), the cephid *Cephus cinctus* (Robertson *et al.* 2018) and the orussid *Orussus abietinus* (Oeyen *et al.* 2020). *Neodiprion*, *Cephus*, and *Athalia* have been sequenced because of their status as pests on pines, wheat, and *Brassica*, respectively, while the interest in the Orussidae follows from its status as the sister taxon to the predominantly parasitic and carnivorous Apocrita (Oeyen *et al.* 2020).

**Figure 1:**
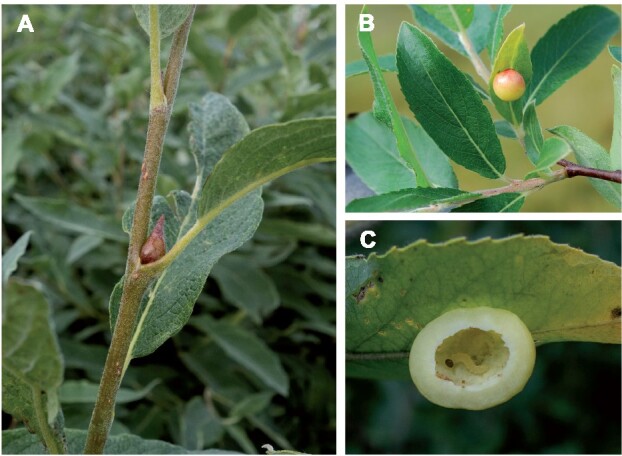
(A) Bud gall induced by *E. lappo* on *Salix lapponum*. (B) Leaf gall induced by *E. aestiva* on *S. myrsinifolia*. (C) Larva of *E. aestiva* inside opened gall. (Photographs by TN).

In order to expand the representation of Tenthredinidae, the most species-rich family within the Symphyta, we sequenced and assembled the genomes of the gall-inducing sawflies *Euura lappo* and *Eupontania aestiva* (*Euura saliciscinereae sensu*Liston *et al.* 2017). These species belong to the subtribe Euurina, a monophyletic and diverse group of nematine sawflies that induce galls on willows (*Salix* spp.). Depending on the species, the females oviposit into the leaves, petioles, shoots, or buds of their willow hosts; plant hormones or hormone analogs injected along with the egg lead to the formation of galls that the larvae feed within (Yamaguchi *et al.* 2012). Of our focal species, *E. lappo* induces bud galls on *S. lapponum* (Figure 1A), while *E. aestiva* produces pea-shaped galls on the underside of leaves of *S. myrsinifolia* (Figure 1, B and C).

The abundance, high species number, marked host specificity, and diverse parasitoid complexes of willow-galling sawflies make them a highly suitable study system for research on host-associated genetic divergence (Leppänen *et al.* 2014) and tri-trophic network ecology (Nyman *et al.* 2007; Gravel *et al.* 2019). In order to facilitate future eco-evolutionary research on Euurina gallers, we utilized a hybrid approach based on 10X Genomics linked-read sequencing, MinION ONT long-read sequencing, and Illumina short-read sequencing (for *E. lappo*) to assemble highly contiguous draft genomes for both focal species. The genomes presented here have a similar level of contiguity and completeness compared to previously published hymenopteran genomes, as inferred from benchmarking universal single-copy orthologs (BUSCO) present in the genomes. Our genomes provide a foundation for future analyses of genomic divergence and adaptation in insect-plant coevolution, as well as expand the representation of symphytan taxa in analyses of phylogenetic relationships and genomic composition within the order Hymenoptera.

## Materials and methods

### Sample collection

Due to the low-input requirements of the library preparation and sequencing strategies that we applied, we were able to use only a single haploid male specimen for each species. The *E. lappo* male (Laboratory ref. # 17059) was collected in Kilpisjärvi, Finland, on August 14, 2016, as a larva within a bud gall on *S. lapponum*. The *E. aestiva* male (Laboratory ref. # TN-EAE_D_712) was collected in Abisko, Sweden on August 18, 2017, from a leaf gall on *S. myrsinifolia*. Both galls were collected in conjunction with more extensive sampling efforts, and the larvae were reared and overwintered as pupae in the laboratory until they emerged as adults in the subsequent spring. Both specimens were stored in 99.5% ethanol at −20°C.

### High molecular weight DNA extraction

High molecular weight (HMW) DNA was extracted from the specimens following an adaptation of the salting-out method of Miller *et al.* (1988) (10x Genomics 2018). Before extraction, the genitalia of the individual males was removed and stored as species vouchers in 99.5% ethanol at –20°C. The remainder of each insect was homogenized using sterile scalpel blades, and then incubated overnight at 37°C in 600 µl lysis buffer (10 mM Tris–HCl, 400 mM NaCl, and 100 mM EDTA, pH 8.0) with 100 µl of Proteinase K (20 mg/ml). Genomic DNA was then salted out by adding 240 µl of 5 M NaCl and cleaned using 70% ethanol. Finally, the extracted HMW DNA was quantified using the Qubit 3.0 system (Invitrogen) and the size distribution (>20 kbp) was confirmed by visualization on a 0.8% Agarose gel alongside a 1Kb extension ladder (Invitrogen).

### Library preparation and sequencing

#### 10X Genomics linked-read sequencing

10X Genomics linked-read sequencing libraries were prepared from 0.5 ng HMW DNA (as determined by an estimated genome size of 270 Mbp) at the Bioscience core lab facility of the King Abdullah University of Science and Technology, Saudi Arabia. The Chromium Genome Reagent Kit v2 provided by the manufacturer was used for library preparation. The final libraries were pooled in equimolar concentrations and then 150-bp paired-end sequenced on a single lane of an Illumina HiSeq4000 platform.

#### Oxford Nanopore long-read sequencing

HMW DNA sequencing libraries were prepared from 400 ng of input DNA using the Ligation Sequencing kit (SQK-LSK109) along with the Native Barcoding Expansion Kit (EXP-NBD104) following the manufacturer’s (Oxford Nanopore Technologies, UK) protocols. The final libraries were then sequenced on a single flow cell (FLO-MIN106D) on the MinION, which was controlled using the MinKNOW version 3.4.8 software. Real-time base calling was turned off and was instead performed on the servers of the CSC—IT Center for Science, Finland, using Albacore version 2.3.4 (Oxford Nanopore Technologies, UK).

#### Illumina short-read sequencing

A whole-genome short-read sequencing library was prepared from 10 ng of *E. lappo* DNA using the NEBNext Ultra II FS DNA Library Prep Kit for Illumina (New England Biolabs, USA) following the manufacturer’s protocols. The library was size-selected at 400 bp and sequenced as part of a pool of samples on a single 150-bp paired-end lane of the Illumina HiSeq2500 platform.

### Genome assembly and validation

#### *Euura lappo* genome assembly

A hybrid *de novo* genome of *E. lappo* v.1 was assembled using 10X Genomics linked reads, raw ONT long sequencing reads, and Illumina short reads with MaSuRCA version 3.3.6 (Zimin *et al.* 2013). The genome was polished using Pilon version 1.23 (Walker *et al.* 2014) by mapping the sequencing reads back onto the assembled genome to correct miss-assemblies and heterozygous sites. The genome was then further scaffolded using Scaff10X version 4.2 (Mullikin and Ning 2003; Murchison *et al.* 2012) with the 10X Genomics reads and Oxford Nanopore reads (assembly version 2.0), followed by a second round of genome polishing with Pilon (assembly version 2.2).

The *E.* *lappo* genome assembly, ELAPPO_v2.2, was validated by mapping the sequencing reads back onto the genome using BWA version 0.7.17-r1188 (Li and Durbin 2009). The mapping rates were then calculated with samtools version 1.4 (Li *et al.* 2009). The contiguity of the assembly was assessed using QUAST version 5.0.2 (Gurevich *et al.* 2013). To validate the assembly size, we compared it against a *k*-mer based genome-size estimate. The whole-genome Illumina sequencing data were used in this analysis, and the optimal *k*-value was determined using KmerGenie version 1.7051 (Chikhi and Medvedev 2014). Jellyfish version 2.3.0 (Marçais and Kingsford 2011) was then used to obtain the frequency distribution of all *k*-mers with length *k *=* *89. The frequency distribution was then analyzed with GenomeScope2 (Ranallo-Benavidez *et al.* 2020) to estimate the genome size and repeat content. Finally, the completeness of the genome assembly was estimated by comparison to the single-copy orthologs from the Hymenoptera_odb10 and Metazoa_odb10 datasets using BUSCO version 4.1.4 (Simão *et al.* 2015; Seppey *et al.* 2019).

#### *Eupontania aestiva* genome assembly

Due to differences in the sequencing strategy for the two focal species, the hybrid genome assembly of *E.* *aestiva* was assembled using 10X Genomics linked reads and raw ONT long sequencing reads with MaSuRCA version 3.3.6. The subsequent steps for genome polishing, scaffolding, and validation, were the same as described above for *E. lappo*. However, the 10X Genomics linked reads and ONT data were used for polishing, and the *k*-mer based estimation of genome size and repeat content was performed using the 10X Genomics linked read data.

### Genome annotation

Repeat annotation was performed using the extensive *de novo* TE annotator (EDTA) pipeline (Ou *et al.* 2019). This pipeline streamlines the identification and classification of repeats, by using commonly used programs, such as RepeatModeler (Smit and Hubley 2015), LTR Finder (Xu and Wang 2007), LTRharvest (Ellinghaus *et al.* 2008), and HelitronScanner (Xiong *et al.* 2014) to create a *de novo* repeat library. RepeatMasker (Smit *et al.* 2013) and the final EDTA repeat libraries were then used to soft mask the genome assemblies prior to annotation.

Gene prediction was completed *ab initio* using the BRAKER2 pipeline (Hoff *et al.* 2019) in conjunction with Genemark-ES (Lomsadze *et al.* 2005) and Augustus (Stanke *et al.* 2006). Functional annotation of the predicted genes was provided by Protein ANNotation with Z-scoRE (PANNZER2) (Koskinen *et al.* 2015).

### Gene homology

The protein sequences of the predicted genes from the two genomes were compared to previously published protein sequences annotated from the genomes of *Acyrthosiphon pisum* (GCA_005508785), *Tribolium castaneum* (GCA_000002335), *Drosophila* melanogaster (GCA_000001215), *A.* *rosae* (GCF_000344095), *C.* *cinctus* (GCF_000341935), *N.* *lecontei* (GCA_001263575), *O.* *abietinus* (GCF_000612105)*, Ceratosolen solmsi* (GCA_000503995), *Nasonia vitripennis* (GCF_000002325), and *Apis mellifera* (GCF_003254395) using OrthoFinder2 version 2.3.12 (Emms and Kelly 2015, 2019). For visualization, the orthogroups were restricted to eight hymenopteran species and graphed using UpSetR (Lex *et al.* 2014; Conway *et al.* 2017).

To determine how our two focal genomes fit phylogenetically with other published hymenopteran genomes, we identified BUSCOs for 13 other hymenopteran species [*A.* *rosae* (GCF_000344095), *N.* *lecontei* (GCA_001263575), *N.* *pinetum* (GCA_004916985), *C.* *cinctus* (GCF_000341935), *O.* *abietinus* (GCF_000612105), *Ormyrus nitidulus* (GCA_900474335), *N.* *vitripennis* (GCF_000002325), *Cecidostiba fungosa* (GCA_900474305), *Ceciostiba semifascia* (GCA_900474235), *Polistes dominula* (GCF_001465965), *A.* *mellifera* (GCF_003254395), *Atta cephalotes* (GCF_000143395), and *Solenopsis invicta* (GCF_000188075)] and one outgroup [*T.* *castaneum* (GCA_000002335)]. Amino acid sequences from 451 BUSCO genes (56,037 amino acid sites), where all focal taxa were represented, were aligned using MUSCLE (Edgar 2004) and trimmed using TrimAl (Capella-Gutiérrez *et al.* 2009). A consensus maximum-likelihood tree was calculated using ModelFinder (Kalyaanamoorthy *et al.* 2017) and IQ-TREE based on the LG+F + I+G4 substitution model (Nguyen *et al.* 2015), and clade support were inferred based on 1000 bootstrap iterations (Hoang *et al.* 2018).

## Data availability

The genome assemblies and sequencing reads are available from GenBank and the SRA databases under BioProject accession numbers PRJNA692175 (*E. lappo*) and PRJNA692828 (*E. aestiva*).

## Results and discussion

### Genome assembly

#### Quality of genome assemblies

The quality of the genomes was first assessed by mapping the sequencing reads back onto the two assemblies. The read-mapping rate was 98.2% for *E. lappo* and 97.1% for *E. aestiva*. In the next step, we utilized two BUSCO databases to estimate the completeness of universal single-copy orthologs. The *E. lappo* genome had 93.5% of the total complete single-copy hymenopteran BUSCOs [(S-Single copy: 91.8%, D-Duplicated: 1.7%), F-Fragmented: 1.5%, M-Missing: 5.0%, n-Total: 5991] and the *E. aestiva* genome contained 90.2% [(S: 88.4%, D: 1.8%), F: 2.2%, M: 7.6%, n: 5991]. The *E. lappo* genome had 96.3% of the total complete single-copy metazoan BUSCOs [(S: 94.9%, D: 1.4%), F: 0.7%, M: 3.0%, n: 954] and the *E. aestiva* genome contained 97.8% [(S: 97.5%, D: 0.3%), F: 1.2%, M: 1.0%, n: 954]. Hence, both methods indicate good assemblies with near-complete hymenopteran and metazoan core gene sets, suggesting that most genes are present in the annotation of our draft genomes.

##### Euura lappo

The assembled genome length for *E. lappo* was 259.85 Mb, which is consistent with the *k*-mer-based genome-size estimate of 248.28 Mb, as well as with lengths of previously published hymenopteran genomes (Robertson *et al.* 2018; Oeyen *et al.* 2020). The assembled genome consisted of 2503 contigs, with 50% of the genome contained in the 329 longest contigs (Table 1).

**Table 1:** Assembly statistics for the genomes of *E. lappo* and *E. aestiva*

	*E. lappo*	*E. aestiva*
10X linked reads coverage	66X	135X
MinION nanopore coverage	9X	10X
Illumina shotgun coverage	169X	n.a.
Total length (bp)	259,850,900	222,225,666
Number of contigs	2,503	16,952
Longest contig (bp)	1,919,081	797,452
GC-%	40.5	40.25
N50	208,956	49,744
N75	102,897	13,796
L50	329	1,156
Complete BUSCOs—count (%)	5,602 (93.5%)	5,404 (90.2%)

##### Eupontania aestiva

The length of the assembled *E. aestiva* genome was 222.23 Mb, which is smaller than the *k*-mer-based estimate of 287.95 Mb. The latter estimate is likely affected by the *k*-mer counting being based solely on 10X linked reads, but both values are nevertheless close to the estimated size of the *E. lappo* genome, as well as to the aforementioned estimates for other hymenopteran species. The *E. aestiva* genome contained 16,952 contigs, and 50% of the genome was contained in the 1156 largest contigs (Table 1).

### Genome annotation

#### Repeat annotation

The EDTA repeat annotation pipeline showed that both genomes contained a large proportion of repetitive elements. The masked repeat proportion of the genome was 27.45% in *E. lappo* and 16.89% in *E. aestiva* (Table 1). For *E. lappo*, the estimate was close to the repeat-element composition based on *k*-mers reported by GenomeScope2 (23.1%), but GenomeScope2 predicted a higher fraction of repeats for *E. aestiva* (44.9%). The difference in the estimated repeat content is likely due to the *k*-mer frequency of the 10X Genomics sequencing data being biased due to the method of library creation. Interestingly, the *E. lappo* assembly contained more gypsy-type LTRs than did the *E. aestiva* assembly (Table 2). Both genomes also contained a much higher proportion of repeat elements than the 4.33% (3.19% as annotated by EDTA) reported for *A.* *rosae* (Petersen *et al.* 2019). The difference is most likely due to our use of long-read sequencing technologies, which allow better assembly of repeat elements as compared to datasets based on only Illumina short reads (Schmidt *et al.* 2020).

**Table 2:** *De novo* repeat annotation of the *E. lappo* and *E. aestiva* genomes

	*E. lappo*			*E. aestiva*		
Repeat class	Count	bp masked	% masked	Count	bp masked	% masked
DNA						
DTA	26,498	7,245,658	2.79	26,790	6,952,423	3.15
DTC	19,198	5,100,000	1.96	14,058	3,592,678	1.63
DTH	2,169	498,037	0.19	359	72,585	0.03
DTM	45,357	11,815,614	4.55	28,874	6,809,103	3.08
DTT	1,480	490,372	0.19	419	128,294	0.06
Helitron	13,267	4,941,840	1.90	20,362	4,941,319	2.24
LTR						
Copia	9,789	4,041,679	1.56	2,175	783,202	0.35
Gypsy	34,191	19,359,338	7.45	5,264	2,092,539	0.95
Unknown	50,728	14,651,949	5.64	36,128	10,241,713	4.64
MITE						
DTA	3,143	592,895	0.23	4,036	729,500	0.33
DTC	1,592	285,828	0.11	1,096	170,108	0.08
DTH	149	23,489	0.01	116	15,614	0.01
DTM	16,660	2,259,373	0.87	5,305	759,185	0.34
DTT	188	32,332	0.01	42	3,311	0
Total	224,409	71,338,404	27.45	145,024	37,291,574	16.89

#### Gene prediction

The number of genes predicted *ab initio* was 23,848 and 24,979 for the *E. lappo* and *E. aestiva* genomes, respectively. We acknowledge that this method likely overestimates the true number of genes present due to false positives (Salamov and Solovyev 2000; Misawa and Kikuno 2010), as many hymenopterans have between 12,000 and 20,000 predicted genes (Branstetter *et al.* 2018), but it nevertheless provides a better understanding of the gene repertoires compared to genomes without any form of annotation. The *ab initio* predicted protein set in *E. lappo* had 86.2% of the total complete single-copy BUSCOs [(S: 84.4%, D: 1.8%), F: 3.2%, M: 10.6%, n: 5991], and the corresponding proportion for *E. aestiva* was 87.9% [(S: 86.7%, D: 1.2%), F: 4.6%, M: 7.5%, n: 5991]. Due to the quality of the genomes, it is likely that the annotation can be improved through the addition of RNA-seq data in the future.

### Gene homology

**Figure 2:**
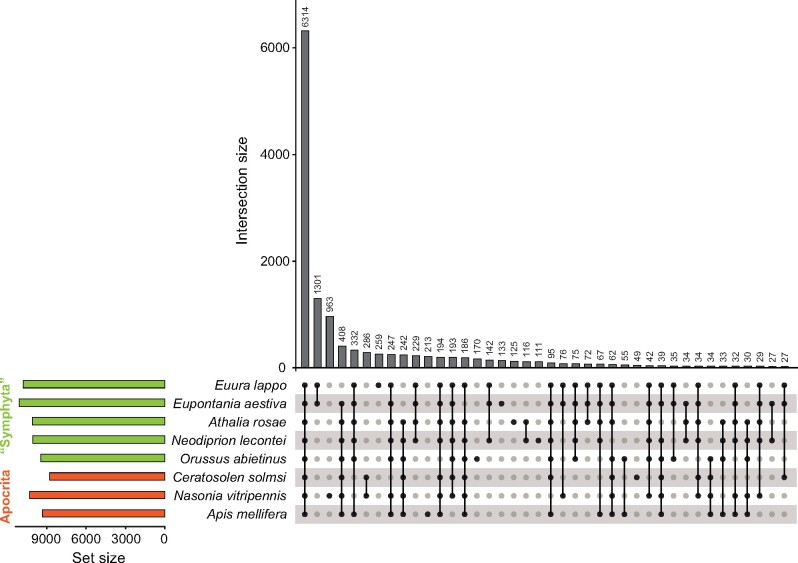
UpSet plot showing the number of orthogroups shared across different partitions of the included hymenopteran protein sets. Set size reflects the total number of orthogroups contained in the protein repertoire of each species, while intersection size indicates the number of orthogroups in common among species or unique to a species. Single dots in the lower panel indicate orthogroups unique to a particular species, and dots joined by lines indicate orthogroups shared across species.

OrthoFinder2 assigned 95.1% of all proteins from the included 12 insects to one of 22,225 orthogroups, with the remaining ones defined as unassigned. The degree of overlap among the included insect species was 4780 orthogroups, which is likely a reflection of the core gene set of these taxa. When the analysis was restricted to only eight hymenopteran taxa (Figure 2), a total of 14,382 orthogroups were predicted. The protein sets predicted from our genomes had a high proportion (*E. lappo* 94.5%, *E. aestiva* 95.0%) of genes assigned to one of these orthogroups. Altogether 6314 orthogroups contained genes from all of the included hymenopteran species, and this likely represents the ‘core’ hymenopteran protein set. The validity of our *ab initio* gene predictions is supported by the fact that the genomes presented here contain >55% of the genes predicted in the recently published *A. rosae* (11,894 genes) and *O. abietinus* (10,959 genes) genomes (Oeyen *et al.* 2020).

**Figure 3:**
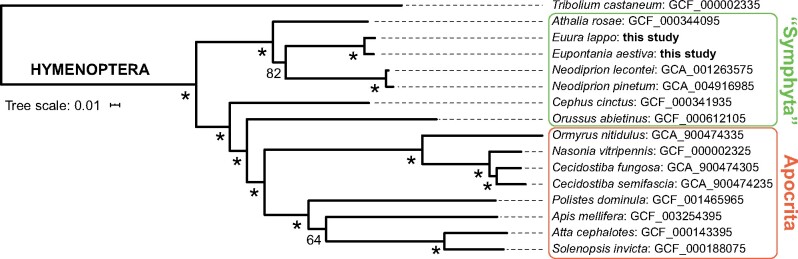
Maximum-likelihood tree of 15 hymenopteran taxa and one coleopteran outgroup (*T. castaneum*) based on amino acid sequences of 451 BUSCOs shared by all focal taxa. Numbers below branches indicate clade support (%) according to 1000 ultrafast bootstrap iterations (* = 100%).

The general structure of the phylogenetic tree estimated on the basis of amino acid sequences of 451 BUSCO genes (Figure 3) agrees with previous phylogenetic (Malm and Nyman 2015) and phylogenomic (Branstetter *et al.* 2017; Peters *et al.* 2017) analyses of the Hymenoptera. The placement of our two focal tenthredinid gall inducers as sister to the Diprionidae (with the exclusion of *Athalia*) is consistent with the combined morphology + sequence data results of Schulmeister (2003), as well as with the recent results of Branstetter *et al.* (2017), which were based on sequencing of ultraconserved genomic elements (UCEs). Interestingly, this topology indicates that our two galler genomes are, in fact, the first representatives of Tenthredinidae *sensu stricto*.

## Conclusions

The genomes of *E.* *lappo* and *E.* *aestiva* presented in this study are of good draft quality, with a contiguity and coverage comparable to previously published hymenopteran genome assemblies. Hence, our study shows that assembling high-quality hymenopteran genomes can be realized using a reasonably small amount of sequencing with only a single 10X genomics linked-read library as well as MinION long-read technology. The genomes presented here also have a higher content of repeats compared to previously published hymenopteran genomes; this is likely due to the better ability of long-read sequencing technologies to sequence through these regions, and suggests that the repeat content of hymenopteran genomes may have been underestimated.

Even though numerous hymenopteran genomes have been published during the last decade, plant-feeding symphytan lineages are still severely underrepresented in genomic databases. This is the case especially for the globally distributed and ecologically diverse sawfly family Tenthredinidae, which includes over 5000 described species (Taeger *et al.* 2010). The genomes presented here are a step towards correcting this bias, and will constitute a highly useful resource for analyses of higher level hymenopteran phylogenetics, development of genomic markers, and elucidation of genome structure and function within the order. In particular, when combined with further data on related species, the genomes of *E. lappo* and *E. aestiva* will enable comparative analyses of the genetic basis of adaptation and speciation in specialist insect herbivores (cf. Leppänen *et al.* 2014). As shown by Yamaguchi *et al.* (2012), adult females and larvae of willow-galling sawflies are able to produce plant hormones or hormone precursors, so our genome data should also help to understand the mechanisms that underlie plant manipulation by gall-inducing insects (*cf*. Korgaonkar *et al.* 2021).

## Funding

This research was funded by the Academy of Finland (project 294466 to T.N.) and by baseline funding from King Abdullah University of Science and Technology (to M.A.).

*Conflicts of interest:* None declared.
